# The state of undergraduate palliative care education at Austrian medical schools – a mixed methods study

**DOI:** 10.1186/s12904-023-01255-9

**Published:** 2023-10-10

**Authors:** Véronique Toussaint, Piret Paal, Rainer Simader, Frank Elsner

**Affiliations:** 1 Department of Palliative Medicine, Medical Faculty RWTH Aachen University, Aachen, Germany; 2 Institute of Palliative Care, Paracelsus Medical University, Salzburg, Austria; 3 Hospiz Österreich / Österreichische Palliativgesellschaft, Vienna, Österreich

**Keywords:** Palliative care, Palliative medicine, Undergraduate education, Teaching, University, Medical school, Austria, Europe, European Region

## Abstract

**Background:**

There is an increasing demand for universal, high-quality access to palliative care in Austria. To ensure this, the implementation of palliative care in the medical studies curriculum is essential. This is the first study to investigate the state of undergraduate palliative care education at Austrian medical schools.

**Methods:**

For this mixed-methods study with concurrent embedded design, expert interviews and online surveys were conducted between March and August 2022. The interviews were subjected to a thematic analysis according to Braun and Clarke, while the questionnaires were analysed descriptively-statistically. For the final integration, the results of both methods for each topic are presented and discussed complementarily. Both the primary qualitative and supportive quantitative data were collected to combine the advantages of the in-depth nature of the qualitative data and the consistent structure of the quantitative data to provide a more precise representation of the state of teaching.

**Results:**

Twenty-two persons participated in the study, of whom twenty-one participated in the interview and eight in the questionnaire. The participants were experts in palliative care teaching at Austrian medical schools. Currently, palliative care is taught at seven out of the eight universities. Large differences were found in the number of hours, organisation, teaching formats, and interprofessional education. At present, three universities have a chair for palliative care and at least five universities have access to a palliative care unit.

**Conclusion:**

Undergraduate palliative care education in Austria is very heterogeneous and does not meet the minimum standards suggested by the European Association for Palliative Care (EAPC) curriculum recommendations. However, several universities are planning measures to expand palliative care teaching, such as the introduction of mandatory teaching or the establishment of new teaching formats. Better coordination and networking within and between universities would be beneficial for the expansion and quality of teaching.

**Supplementary Information:**

The online version contains supplementary material available at 10.1186/s12904-023-01255-9.

## Background

Palliative care is increasingly gaining importance in Austria, and since 2022, two new laws that provide momentum for the expansion of palliative care have been in effect. On the one hand, the Hospice and Palliative Fund Act (*Hospiz- und Palliativfondsgesetz*) regulates funding throughout Austria and promotes Palliative Care in the form of special-purpose grants [1]. On the other hand, medically assisted suicide is now possible under certain conditions through the Dispositions of Dying Act (*Sterbeverfügungsgesetz*) [2]. A joint statement by the two Austrian organisations for Hospice and Palliative Care, *Hospiz Österreich* and *Österreichische Palliativgesellschaft,* explicitly points out that assisted suicide must not be the only alternative for patients and their relatives to cope with difficult situations at the end of life. Instead, nationwide high-quality access to palliative care is required [3, 4].

According to the World Health Organization, the aim of palliative care is to improve the ‘quality of life of patients and their families facing the problems associated with life-threatening illness through the prevention and relief of suffering’. It prevents and treats ‘physical, psychosocial, and spiritual’ symptoms and is ideally integrated at an early stage of the illness [5]. To best respond to this objective, palliative care should take place in an interprofessional team so that people from different professional groups can work together to improve the quality of care [6–9].

The expansion of palliative care has been discussed both nationally and internationally. Health professional education is considered a central approach [3, 4, 10–14]. A recent WHO paper even shows that education and training are a fundamental wall of the ‘conceptual model of palliative care development’ [15].

At the postgraduate level in Austria, there is a specialisation for palliative care available since 2017 [16, 17] and a broad range of palliative care courses has been developed in recent years, to which many professional groups have responded positively [9, 18, 19]. However, people with palliative needs are not only treated in specialised palliative care facilities. According to a recent Austrian study, they account for 10% of the patients on emergency admission [20]. Demand for palliative care treatment is also increasing due to demographic developments, which include a rise in cancer prevalence [21] as well as in the over-60 group [22].

In this respect, basic palliative care knowledge, skills and attitudes should not be taught only to physicians who choose to pursue postgraduate palliative training or the specialisation. Instead, all physicians should be able to provide basic palliative care. Several international studies have shown that, many young physicians often feel inadequately prepared for this task during their medical studies [23–25]. Furthermore, it has been found that medical students benefit greatly from palliative courses in their undergraduate studies in terms of their competence and well-being in later treating patients with palliative care needs [24, 26–31]. As a result, the authors of these studies highly recommend the integration of palliative care in the undergraduate curriculum. This is in accordance with the suggestions of the European Association for Palliative Care (EAPC), which has called for the implementation of palliative care teaching in medical studies for many years and has developed several curriculum guidelines for this purpose [32–34].

To advance undergraduate education and, thus, the expansion of palliative care in Austria, it is important to understand the current state of teaching. However, this has hardly been investigated in Austria thus far, as a recently published scoping review shows [35]. Therefore, in light of the two new laws in effect, the aim of this mixed-methods study using interviews and questionnaires is to assess the state of undergraduate palliative care education in Austrian medical schools for the first time.

## Methods

The present study consists of a mixed methods study with an embedded concurrent design, as it combines two data sets that were collected simultaneously using different methods. Following the work of Kettles et al., the data set that contributes to a higher proportion of the reported outcomes is referred to as primary data, while the other data set collected with a more supportive role is referred to as secondary data [36]. The primary data is qualitative and is gathered through semi-structured expert interviews on the state of undergraduate palliative care education in Austria as well as on background information and perceived influencing factors, barriers, and opportunities. The secondary data set, which is quantitative, was obtained through a closed web questionnaire on the palliative care teaching at Austrian universities. The advantage of this methodology lies in combining the benefits of both the depth of the primary qualitative data and the consistent structure of the secondary quantitative data, thus providing a more accurate picture of the state of teaching. An embedded design was chosen because of the emphasis on the primary qualitative data. Data collection took place from March to August 2022.

### Participants

To be eligible to participate in our study, respondents should be ‘experts’ in undergraduate palliative care education in Austria. In general, ‘experts’ are assumed to have special knowledge and specific functions similar to societal ascription [37]. For our study, we aimed to identify and recruit key persons who are either involved in or responsible for palliative care teaching at an Austrian university or have an external overview of palliative care education in Austria.

Regarding the sample for the primary qualitative data, several aspects must be considered to obtain sufficient information power, according to Malterud et al.[38]:

- Broad study objective: to survey the state of undergraduate palliative care teaching
- A cross-case outcome analysis: coverage of teaching at all universities in Austria
- High specificity of the sample through purposive sampling: recruitment of the best-informed key persons from all medical universities in Austria
- Theory-based study: extensive literature review, for example, in the context of the scoping review [35], established interview guide (see below), and high competence in qualitative analysis in the team of authors
- Variable quality of dialogues, which according to field notes and transcripts was predominantly positive

Considering these points, a sample of two key persons per university and between two and three external key persons was aimed for the expert interviews to obtain sufficient information power.

For the completion of the questionnaire, we decided to invite one participant per university who was presumed to be the best-informed key person in palliative care teaching at the respective university. This person could also be part of the interview sample. It was allowed and encouraged that this key person consults their colleagues in case of uncertainty. Participation was limited to one person per university to ensure the representativeness of the responses, while also preserving the participants' anonymity.

For the identification of the key persons at the universities, a representative of the Austrian association *Hospiz Österreich* contacted the rector's or dean’s university offices asking which persons were most familiar with the organisation and realisation of palliative care teaching at the respective university. External experts were primarily identified via the network of the Austrian association *Hospiz Österreich.* In addition, further representatives of universities and external experts were identified through research by the authors and also during the study through mentions by participants who had already been interviewed. The recruitment of the participants was carried out via e-mail. Considering the limited time resources of our participants, recruitment for the interviews and the questionnaires was carried out simultaneously to reduce the number of contacts and avoid drop-outs.

### Primary data: qualitative data obtained through semi-structured expert interviews

The format of the semi-structured expert interviews was chosen to collect the qualitative data. For this purpose an interview guide with open-ended questions was generated, which ‘serve[d] to guide, but not constrain’ the interview [39]. The course and topics of conversation varied between participants in consideration of their individual knowledge and background. The interview guide (see Additional File 1 for a translated version) was developed based on a comparable study in China [40] and adapted to Austria after extensive literature research. It was reviewed several times by all the authors involved in the study. Internal testing was conducted through a mock interview and an external expert assessment by a professor of psychology experienced in qualitative research. Field testing of the interview guide could not be conducted due to the small number of experts in Austrian undergraduate education. The interview guide was slightly adapted during the study, in the nature of an iterative process [41].

The interviews were conducted by the corresponding author VT. At the time of data collection, she was a Luxembourgish medical student at a German university in her 6th year. Before the start of the study, she had no experience in interviewing or qualitative analysis, but was individually trained for this by the co-authors FE and PP. The interviewer had no relationship with the participants prior to the start of the study. Before the interviews, the participants received a cover letter and an informed consent form so that they were aware of the background of the interviewer, as well as the study objective, methodology, data protection requirements, and the desire to publish the collected results.

Due to the Covid 19 pandemic, interviews were conducted via the digital platform Zoom. This allowed for more flexibility in the scheduling and collection of high-quality audio-visual recordings.^1^ During the interview, the participants were mostly in offices, doctors' offices, or private surroundings. No other people appeared to the interviewer during any of the interviews. The interviews were conducted in German, the interviewer's native language and the native or working language of all participants. Immediately after each interview, the interviewer took notes on the setting, atmosphere, feelings triggered by the interviews, and interview performance.

### Secondary data: quantitative data obtained through a closed questionnaire

The quantitative survey consisted of a closed questionnaire, as only a selected sample had access to it [42]. The presumed best-informed key person from each university received an invitation by e-mail to complete the questionnaire. Participants were given the opportunity to study the questionnaire in advance. The questionnaire (see Additional File 2 for a translated version) was developed based on previous comparable studies from other countries [43, 44] and adapted for Austria after extensive literature research. It contained 29 questions on teaching infrastructure, organisation, content, and formats as well as examination content and formats of palliative care at the respective university. The format of the questions consisted mainly of multiple-choice or dichotomous selection questions, some of which were supplemented with empty text fields for open answers. The questionnaire was implemented using the online tool SoSci Survey, which is free for non-commercial research [45]. In order to reduce the length of the questionnaire, adaptive questioning was used so that, for example, more detailed questions on mandatory teaching only appeared if it was ticked that mandatory teaching was offered at the respective medical school. The online survey contained between one and four items per page and between seven and 17 pages. The order of the items was not randomised. It underwent a technical functional test, pre-test, and multiple checks, following the steps described in the checklist CHERRIES that set the standard for reporting online surveys [42]. To ensure that the questionnaire was completed only once per university, it was sent to key persons by e-mail via a personalised link that was only valid for one successful participation. Informed consent was obtained from the first page of the questionnaire and was approved to proceed with the questionnaire. Respondents had the opportunity to review and change their answers as well as to pause the questionnaire and continue filling it out later.

### Analysis

Data processing and analysis of the interviews was carried out using the MAXQDA 2022 Analytics Pro software [46]. The interviews were transcribed by the corresponding author. The initial codes, representing the smallest possible functional units of the transcripts, were then generated for the entire dataset. This was performed inductively, that is, based on the available data. The codes were then divided into themes and sub-themes. These steps were repeated several times in the sense of a ‘recursive process’ in which codes and themes were repeatedly changed and adapted. In the thematic analysis, 1625 segments were coded, and 10 main themes were identified. The analysis was conducted in a ‘contextualist method’, thus looking objectively and realistically at the reproduced content, but also considering the information ‘underneath the surface’ and the ‘larger social context’ [47]. The transcripts were pseudonymised for thematic analysis and anonymised in a second step for this article and further archiving.

Using the SoSci Survey tool, the questionnaires were anonymised and analysed descriptively-statistically using Excel by the corresponding author. For this paper, we selected a subset of questions that we considered informative and relevant. For example, the question of how long palliative care has been taught at the respective university was excluded, as this question is inextricably linked to the age of the medical faculty and therefore cannot be assessed independently of it. Further questions were omitted in order to protect the anonymity of the participants^2^ and because of a low response rate to certain questions.^3^

#### Integration of qualitative and quantitative results

For this mixed-methods study with embedded concurrent design, primary qualitative data and secondary quantitative data were collected simultaneously. We opted for a concurrent approach because this allowed us to contact the key persons only once, as mentioned above, and, given the dynamic developments in the field, to obtain complementary information at a similar time.

After an initial separate analysis, as described above, the research results were considered and discussed together. When comparing results of two different methods, it is not unusual that they contradict each other on the one hand and explain each other on the other hand [48]. By complementing both methods, the data can convey information that is broader, deeper, and closer to the truth than individually. In this respect, it was decided to present quantitative and qualitative results for each topic together and complementary to each other. For this purpose, the selection and the structure of the initially inductively determined topics in the interviews was adapted to the items of the questionnaire. The outline of this paper therefore follows the selected subset of the questionnaire and adds information from the interviews to each item. By presenting the questionnaire responses and the corresponding interview statements simultaneously, the differently generated information about the state of palliative care education is directly compared and supplemented with background information derived from the experiences and perspectives of the interviewees. As a result of this outline, and for better readability, it was decided that the additional themes identified in the interviews, such as perceived influencing factors, barriers, and opportunities, would not be further elaborated in this paper, but would be addressed in a later publication.

The data collected from the questionnaire and interview statements were translated into English for this article. In addition, the language of citations was cleaned for simple transcription [49]. A supplementary table with quotes on the original language may be available upon reasonable request.

To report the results of qualitative exploration, the checklist Standards for Reporting Qualitative Research (SRQR) [50] and Consolidated Criteria for Reporting Qualitative Research (COREQ) [51] were consulted.

## Results

The following eight medical faculties in Austria (sorted by age) could be identified in advance:

- Medical University of Vienna [52]
- Medical University of Innsbruck [53]
- Medical University of Graz [54]
- Paracelsus Medical University (Salzburg) [55]
- Karl Landsteiner University of Health Sciences (Krems) [56]
- Johannes Kepler University Linz [57]
- Sigmund Freud University of Vienna [58]
- Danube Private University (Krems) [59]

### Participants

In total, 22 of the 26^4^ invited key persons participated in the study. Of these 22 key persons seven participated in the interview and in the questionnaire, one participated only in the questionnaire, and fourteen participated only in the interview.

The questionnaire was completed by eight university representatives (one person per university). This corresponded to a response rate of 100%. Seven of these participants indicated that they were physicians and had either advanced training in palliative care or a specialisation in palliative care. One participant was an employee of the university's business administration department.

Twenty-one interviews were conducted, each with a recorded duration of 24–45 min. In addition to the four external experts, one to three key persons per university were interviewed. More detailed information about the interviewees can be found in Table 1.

**Table 1:** Socio-demographic characteristics of the interviewees

Category	Number	Percentage
**Gender**		
Female	12	57.1%
Male	9	42.9%
**Relation to undergraduate palliative care education in Austria**		
Current occupation in undergraduate palliative care education	14	66.7%
Current occupation in undergraduate non-palliative care education	1	4.8%
Former occupation in undergraduate palliative care education	1	4.8%
Future occupation in undergraduate palliative care education	1	4.8%
External experts	4	19.0%
**University degree**		
Professor	8	38.1%
Private lecturer (*Privatdozent*)	2	9.5%
No post-doc (*Keine Habilitation*)	11	52.4%
**Profession**		
Physician	21	100.0%
**Clinical work environment**		
Palliative care unit (Austria)	10	47.6%
Hospice (Austria)	3	14.3%
Palliative care unit (other country)	3	14.3%
Retirement (after palliative care work in Austria)	2	9.5%
Non-palliative care unit (Austria)	1	4.8%
Non-palliative care unit (other country)	1	4.8%
Medical directorate (Austria)	1	4.8%
**Total**	21	

### Outline of the results

This paper addresses both the system associated with education, such as chairs, access to a palliative care unit and organisation, as well as teaching itself. In the investigation of teaching, the focus is placed on mandatory education, as this occurs at many more universities than elective teaching. In addition, the eventual implementation of interprofessional teaching and clinical practical year are presented.

### Structures

#### Chair

An Internet search yielded references to a total of three professorships for palliative care in Austria, including two chairs and one endowed professorship: the Medical University of Vienna [19, 60], the Paracelsus Medical University [19, 61], and the Sigmund Freud Private University of Vienna [62].

The information found on the Internet was confirmed in the interviews, but not in the questionnaires, where only participants from two universities ticked that there was a professorship for palliative care at their university. The relevance of a chair for palliative care was emphasised in numerous interviews. Many said that it was important to improve teaching and research at their respective universities. At the same time, the existence of only three professorships was perceived as unsatisfactory.

“Well, of course it would be desirable if we had a chair for palliative care […] because then the value of palliative care would increase […] and if you could then be even more involved in the curriculum, you would also have time for it. So that would be something that, I think, could improve teaching and research in the future.” (Transcript (T) 7, Item (I) 79–81).

“I miss the chairs [for palliative care] in Austria. I would like to have more.” (T 11, I 81)

#### Access to a palliative care unit

In the questionnaire participants were asked which structures or institutions were involved in the teaching of palliative care at the respective university. The detailed answer to this question is provided in Table 2. In summary the questionnaire indicated that access to a palliative care unit existed in five of the eight universities. The results of the interviews largely corresponded with those of the questionnaire. Representatives of the universities without access to a palliative care unit saw this circumstance as a major problem for teaching, since it meant that students could not have contact with patients and could only conduct palliative care research to a limited extent.

"There's no such structure that conducts clinic and research, and that is associated with the university. And you can just notice this deficit.” (T 8, I 55)

“Yes, unfortunately it [practice-oriented teaching] is difficult, of course, because […] there is no palliative care unit” (T 8, I 79)

**Table 2:** Structures affiliated to the universities

Structures affiliated to the university	Statements of each of the eight Austrian universities^a^								Total
Palliative care unit in the affiliated university hospital	X				X		X	X	4
Palliative care unit in a teaching hospital of the university	X			X				X	3
Hospice			X			X			2
Mobile palliative team		X		X			X	X	4
Other: “Palliative Consultation Service”				X					1
Other: “anesthesia, intensive care, emergency medicine and pain therapy”			X						1

^a^The order of the questionnaires was randomised for each table separately

In the interviews, the lack of palliative care units in Austria in relation to the large supply of hospices was mentioned as one of the main obstacles to the development of palliative care education in recent years.

“In the past, no one at the university was interested [in palliative care], and the hospices more or less took it over. And they also do most of the training of physicians and nursing staff. […] I think that's also one of the reasons why this distinction between hospice medicine and palliative medicine doesn't work, because 90% of the training that is done is through hospice institutions. And that […] [a palliative care unit] should actually be a throughput unit like an intermediate care unit, precisely for palliative patients, and dying can also take place somewhere else—that is unfortunately not in people's minds. And if you bring that back into people's minds, then maybe a […] university hospital will be more interested again.” (T 13, I 17).

### Mandatory palliative care teaching

The interviews revealed that six universities had mandatory palliative care teaching. At another university, there was already a voluntary course at the time of the survey that was to be introduced on a mandatory basis in the next few years. At another university, the degree program in human medicine was still so new that in the summer semester of 2023, for the first time, a cohort would reach the clinical section in which palliative care was to be integrated on a mandatory basis. In this respect, all eight universities were expected to offer mandatory palliative care courses in the near future.

In the questionnaire, representatives of seven universities stated that palliative care was taught as a mandatory subject in the academic year 2021/22. For this reason, seven completed sub-questionnaires were available as a basis for the following more detailed presentation of mandatory palliative care teaching.

#### Organisation of the mandatory teaching

**Fig. 1:**
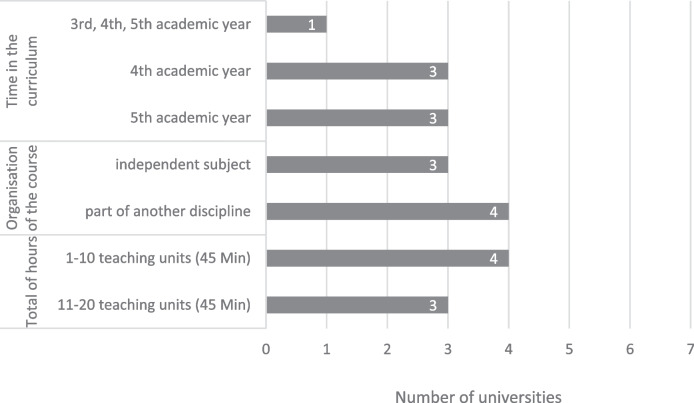
Organisation of the mandatory teaching

Information from the questionnaire on the time, number of hours, and organisation of mandatory palliative care teaching is shown in Fig. 1. The interview statements corresponded to these findings. What was striking in the interview was the wide range of the scope of hours, which was stated as between two and 15 h. At all universities, the purely palliative care courses were planned and held independently by palliative physicians but were embedded in a broader context in which other subjects were also taught. The subjects mentioned were anesthesia, geriatrics, oncology, or a combination of various clinical subjects, for example, in preparation for the clinical practical year. In the interviews, the impression emerged that palliative care played a minor role in the curriculum compared to other disciplines at most universities.

“Palliative care received a part of all this, but that happens only once [during the medical studies], so not for the whole semester” (T 5, I 57)

“We are part of a module, which is actually organised by [another subject]” (T 20, I 49)

In addition, according to the interviews, palliative care aspects also occurred in the courses of other medical disciplines. This served to raise awareness in the first years of the study and later to repeat it regularly.

Many interviewees were content with the allocation of teaching time, especially from the point of view that also due to interdisciplinary teaching, students were repeatedly confronted with palliative care. Interdisciplinary collaboration was positively emphasised at some universities, for example, in the form of seminars and elective courses organised jointly with other subjects. Elsewhere, however, it was criticised that lecturers from other disciplines taught palliative care content without any additional qualifications in palliative care or sufficient coordination with the palliative team.

In the interviews, it appeared that participants had different views on how many teaching hours were required, regardless of the number of hours actually taught. Many wished for more teaching hours, but at the same time noted that, on the one hand, there was limited space in the schedule, and, on the other hand, there was a lack of human resources to offer more teaching in palliative care as those who were responsible had to divide their time between clinical tasks and teaching.

“But the time frame is simply far too short. […] You can really only teach them the basics.” (T 13, I 27)

“It certainly fails because of the time resources […]. But yes, of course you can't (laughs briefly) make the studies twice as long.” (T 2, I 80)

“A few years ago, the rector offered me the following: Let the hospital give you 20 h off per week and in return 20 h for the university—20 h for the clinic […]. That was simply not possible, because that is then missing in the daily clinical routine, and there we have to work on getting more staff again. And then, I think, a lot would be possible. Also in research.” (T 7, I 91)

“The unfortunate aspect of teaching is that it is always done as a side job. I think that is something that should be encouraged in general, that fixed times for teaching should also be established for physicians. [Currently,] it's always secondary. It's always second row, which is not good, actually.” (T 19, I 95)

#### Contents of the mandatory teaching

In the EAPC curricular recommendations for undergraduate education in palliative care, the required content is classified into six categories [32, 33]. In this study’s questionnaire, participants were asked to rank the EAPC categories according to mandatory teaching at their university. Table 3 shows the responses.

**Table 3:** Weighting of the content categories of the mandatory teaching

**EAPC content categories** (with recommended percentage) [32, 33]	**Percentages for each of the eight Austrian universities**^a^								**Mean value**^b^
Basics of Palliative Care (5%)	20%	20%	20%	20%	40%	5%	10%	No mandatory teaching	19.3%
Pain and symptom management (50%)	20%	40%	30%	40%	20%	5%	65%		31.4%
Psychosocial and spiritual aspects (20%)	10%	10%	10%	5%	10%	5%	10%		8.6%
Ethical and legal issues (5%)	20%	10%	10%	15%	10%	40%	15%		17.1%
Communication (15%)	20%	10%	10%	10%	10%	40%	0%		14.3%
Teamwork and self-reflection (5%)	10%	10%	20%	10%	10%	5%	0%		9.3%

^a^The order of the questionnaires was randomised for each table separately

^b^In calculating the mean value, the university where there is no mandatory teaching was not taken into account

The contents mentioned in the interviews are discussed below. Among them, all EAPC categories were also represented.

First, many mentioned the importance of teaching the basics of palliative care. This also included helping students understand the relevance of this subject and eliminating the misunderstanding that palliative care was only care for dying (*Sterbemedizin*). Furthermore, it should be made clear that patients with both oncological and non-oncological life-threatening diseases required palliative treatment.

“I think that undergraduate teaching in palliative care is incredibly important, that you have to plant a seed early on, that students know what it actually means and that there are no longer these retro people, somehow still believing that palliative care means turning up the morphine syringe driver and putting them in bed and letting people die.” (T 5, I 97).

“We try to explain that there are obviously also cardiomyopathies and […] COPD. At some point, [these patients] will need palliative care, unless they get a lung transplant if they are still young, but a patient with massive dyspnea who does not get a lung transplant is clearly a palliative patient. And the exact same thing with cardiomyopathy. And it's the same with neurological diseases, [for example] with stroke symptoms” (T 13, I 59).

Emphasis was placed on symptom and pain control. Explicitly named in this context were the management of pain, dyspnea, nausea, pruritus, constipation, sleep, anxiety, delirium, depression, nutrition, cachexia, sarcopenia, and fatigue. Many interviewees remarked that the management of these symptoms was also of high importance in non-palliative situations and should, therefore, be given sufficient priority in medical education. Additionally, teaching should include the dying process, death, palliative sedation, and end-of-life care.

“It is also good if the students get to know palliative care as early as possible, giving them much more: because these are basic medical skills, and also medically competent skills of symptom treatment, which can always be used, not only now at the end of life, but in general. So you can make palliative care a little more attractive by saying: Wow, they really know how to treat the worst itch or how to deal with pain” (T 5, I 79).

Another concern of many interviewees was the knowledge of the various care structures in Austria, such as hospices, mobile palliative teams, nursing homes, and care assistance. Places in nursing homes and hospices were scarce, and care at home often could not be provided by relatives alone, especially in urban regions. In this respect, students should be familiar with different support options and be aware that patients should not be discharged from the hospital until further care was ensured.

Legal foundations were an important component of undergraduate palliative care education. For example, students should know and understand terms such as patient directive (*Patientenverfügung*), health care proxy (*Vorsorgevollmacht*), and legal representative (*gesetzliche Vertretung*). Some interviewees were also planning to discuss the new Dispositions of Dying Act with students.

The categories of ethics, communication, and self-reflection were frequently associated with the term ‘attitude’ (*Haltung*). It was said to be important to prepare students for ethical questions, such as treatment decision-making and patient autonomy, as well as for difficult conversations with patients or relatives. Furthermore, students should be sensitised to being mindful of themselves and their environment and to realise the significance of palliative care as a comprehensive treatment approach within a continuity of therapy.

“[Nowadays in medicine] we often forget this holistic view and this attitude or this patient-centered work, like: […] What does this patient need, what does he want from me, how can I best help him and when should I stop with therapy?” (T 19, I 77).

“It's an attitude that you just want to give them, like, 'how do I approach my counterpart', also like, 'what do I perceive'” (T 15, I 58)

“Palliative care is part of an overall treatment, a holistic concept” (T 10, I 73).

In particular, external experts noted that it would be useful if there was a structured offering at the university to talk about end-of-life issues and not leave students alone with their experiences and thoughts.

“So I think it is especially about […] in which way students—when they have [been] in their internship and have experienced a lot there—are also picked up by the university and asked: How was it? […] Serious illness, incurability and end of life, caring for relatives, I think these are important questions that are always impressive and that there then is a structured offer to talk about it and not only to share what has been experienced, but also to learn […] I think that would make perfect sense.” (T 16, I 27).

Since most of the work in palliative care takes place in an interprofessional team [6–9], teamwork is discussed under the heading ‘interprofessional teaching’.

#### Teaching and assessment formats and areas

Table 4 contains the individual statements of the participants in the questionnaire regarding the teaching and examination formats as well as the taught and examined areas.

**Table 4:** Teaching and assessment formats and areas of the mandatory teaching

	**Statements of each of the eight Austrian universities**^a^								**Total**	**Proportion**^b^
**Teaching formats**										
Lecture	X	X	X	X	X	X	X	No mandatory teaching	7	87.5%
Bedside teaching	X	X		X		X			4	50.0%
Patient presentation		X	X	X		X			4	50.0%
Practical training	X		X	X					3	37.5%
E-Learning			X^c^	X					2	25.0%
Seminar		X				X			2	25.0%
Problem-based learning	X			X					2	25.0%
Case-based learning				X		X			2	25.0%
Training with simulation patients		X							1	12.5%
**Taught areas**										
Knowledge	X	X	X	X	X	X	X	No mandatory teaching	7	87.5%
Attitude		X	X	X		X	X		5	62.5%
Skills		X		X		X			3	37.5%
**Examination formats**										
Written exam with multiple choice	X	X		X	X	X	X	No mandatory teaching	6	75.0%
Oral exam	X								1	12.5%
Immanent examination			X						1	12.5%
**Examined areas**										
Knowledge	X	X		X	X	X	X	No mandatory teaching	6	75.0%
Attitude		X	X	X		X	X		5	62.5%
Skills		X		X					2	25.0%

^a^The order of the questionnaires was randomised for each table separately

^b^In calculating the proportions, the university where there is no mandatory teaching was also taken into account

^c^Note 'in lockdown'

#### Areas of mandatory teaching

In the questionnaire, the designation 'knowledge' was reported as part of the teaching at all universities and as part of the examination at six of seven universities. Thus, this area is the most common area in mandatory palliative care teaching. This correlates with the reported frequency of lectures in teaching and multiple-choice examinations in assessment.

'Attitude' was stated as an area of both teaching and examination at five of the seven universities with mandatory education. The relevance of attitude as a central learning goal in palliative care teaching was mentioned many times in the interviews and was linked to a holistic and patient-centered understanding of palliative care.

“[Teaching palliative care at the university] means to me above all to convey an attitude that the students do not only understand the medical profession as a scientific art or a technical art, but also […] that the care is really conveyed holistically. Yes, this is about this philosophy and otherwise, above all, making it clear that palliative care is a highly qualified complex treatment. That you don't have this fear that someone is being shunted off to die.” (T 10, I 87).

'Skills' were taught at three universities and examined at two universities, according to the questionnaire. In the interviews, the participants often referred in this context to practice-oriented small-group teaching or practical training, respectively, bedside teaching.

#### Teaching formats of mandatory teaching

According to the questionnaires and interviews, mandatory lectures were held or planned at all universities. Even though this format was predominantly very frontal, some interviewees told what methods they used to make the lectures as engaging and interactive as possible. Examples included bringing in case reports with digital voting devices, telling patient narratives, or discussing medical comics.

“I have case reports and I do them interactively with voting devices. That means patient cases are talked through and […] there are questions about them and of course every question is discussed. […] Naturally I hold my monologues first, so that the basic knowledge is conveyed, but then it continues practically with the cases.” (T 12, I 25).

“I avoid showing any structure or instructive slides. I only show very briefly which facilities there are in Austria, how they are graduated and apart from that I tell patient stories […] These convey much more than anything else in the world” (T 9, I 37–39).

“I think you have to [approach it] with creative methods, like we're trying to do now with the medical comics, where we're putting a lot of effort into using those in the courses to teach very challenging content as well” (T 5, I 89).

Mandatory teaching was complemented by small-group teaching and seminars at several universities. Some interviewees felt that students’ interests were most likely to be aroused in the context of these courses. Moreover, this format was suitable for making teaching more practice-oriented and interactive on the one hand and for discussing particular key contents in more depth on the other. At several universities, case reports were shown, discussed, and/or presented by the students.

“Yes, the seminar is […] a small group lesson, there is a lot of interaction, […] case reports are brought up and then discussed with the students, pros and cons are pointed out and this is just a process of working it out together with the students” (T 2, I 13).

“Sometimes you have teaching courses where no one says anything and you think or you can see that people are playing on their laptops […] and in the seminar I notice that at the beginning it's the same as in all seminars: someone always comes later […] and it's a hustle and bustle and then at a certain point there is absolute silence, so that there is concentration and we always interpret that as interest” (T 6, I 23).

“I am always surprised by how well they familiarise themselves with such a thematic area during the seminar. […] You get the feeling that there is a profound desire to absorb those topics.” (T 10, I 13).

Many interviewees felt that practical training and bedside teaching were important because they would give students a deeper understanding of palliative care and better prepare them for the medical profession later in life.

“In theory, everything always sounds completely different than it is in the end for the individual patient.” (T 20, I 19)

“So, I don't think that the frontal lecture is a good tool for this topic. […] Instead I think it is very important that people come into contact with each other, directly.” (T 1, I 90)

At some universities, students had the opportunity to spend a few hours on a palliative care unit as part of mandatory internships, answering questions, and interviewing a patient or relative, if possible. At one university, a respective internship day was offered in a day hospice.

“[It is] our goal that every student has seen a palliative care unit once, that means a practical training at a palliative care unit with interdisciplinary case discussions about the patients.” (T 19, I 4).

Other participants expressed concerns about the contact with patients. In their opinion, bedside teaching was not suitable for every patient in this difficult phase of life. At one university, where practical training on a unit had previously been offered, it had been noticed that the availability of suitable patients was too variable to ensure consistently comparable bedside teaching. Therefore, it had been decided to replace practical palliative care training with interactive small-group teaching. At another university, palliative care bedside teaching was generally rejected out of consideration for patients. Instead, case reports should be discussed in lectures or seminars, with patient consent.

“I don't want to drag the patient down during rounds. […] You can show things to the student, […] but you shouldn't discuss cases […] at the patient's bedside, […] I don't think much of that.” (T 12, I 57).

At some universities, bedside teaching could not be conducted because there was currently no access to a palliative care unit. The interviewees of the respective universities expressed regret about this on several occasions, as it resulted in a lack of an important component of teaching.

“This means that we can only offer theoretical lectures, which is a pity, because I believe that palliative care would benefit greatly from bedside teaching. Which is of course also a huge problem: as I said, we don't have a palliative care unit.” (T 13, I 3).

While many interviewees emphasised the benefits of practice-based and interactive teaching, others also warned that sufficient time should be allocated to teach theoretical knowledge and for students to study on their own.

“You can't just have lectures from morning until evening, it just doesn't work. I think the mix is quite good with lectures and a lot of practical training. Well, that wasn't the case in my studies. […] I went into work very theory-oriented, so I thought at the beginning: I didn't study at all. (laughs) Because then you were suddenly confronted with problems, which you could solve theoretically, but not practically.” (T 2, I 82).

“They often build a lot on active formats and here you have a seminar, and there problem-based learning, and there again physical examination, and there again the simulation patient and this and this and this. And sometimes I think, students don't have enough time for studying by themselves.” (T 21, I 71).

#### Assessment formats of mandatory teaching

In the interviews, as well as in the questionnaire, it emerged that the most frequently chosen format was the multiple-choice exam. According to the interviews, this corresponded to a cumulative examination at most universities, in which palliative care was represented only in a small to very small proportion. The interviewees revealed that, at almost all universities, palliative care did not have to be passed individually. Interviewees from only one university told of a cross-semester oral examination in which palliative care was one of the subjects that students could be assigned and then had to pass individually.

“Well, you won’t fail if you can’t answer the palliative questions. (laughs)” (T 3, I 167)

However, it was noted in several interviews that multiple-choice exams were not considered an appropriate format to teach palliative care with long-lasting results. While some interviewees generally did not consider an exam to be an appropriate tool for conveying palliative care basics, others proposed alternative assessment formats, such as reflection reports. This was already being planned at individual universities or implemented as a part of interdisciplinary courses*.*

“It’s not about the exam there, it’s about saying, okay, this topic just interests me, right?” (T 12, I 9)

### Other aspects of palliative care teaching

#### Mandatory teaching versus elective teaching

Many interviewees favoured mandatory teaching because it was important to raise awareness of palliative care among all students. This would create a better understanding of the often-misunderstood discipline and possibly motivate more students to pursue palliative care research or to later specialise in palliative care. In addition, the content taught in palliative care was relevant to almost all medical specialties. Several lecturers interviewed reported that there was very high interest among students if this fact was made clear to them.

“At the beginning, they [the students] always say, ‘Yeah palliative…. – I don’t want to become a palliative physician’, but that is always the first thing I say in the lecture: that no matter what specialty they then do later, in every department there are palliative patients that you then have to care for. And I think that often (laughs) the attention is then suddenly higher.” (T 2, I 15).

Other interviewees highlighted the benefits of elective teaching. On the one hand, what was learned voluntarily was often absorbed much more intensively; on the other hand, learning success depended on the personal interest of the students. This interest could not be forced by mandatory teaching and was higher in elective than in mandatory teaching.

#### Elective palliative care teaching

An elective course was offered at three universities according to the questionnaire and at two universities according to the interviews. At the two universities mentioned in the interview, there were frontal elective courses, in which palliative care knowledge was taught in greater depth than in the mandatory curriculum, and practice-oriented teaching formats with a focus on communication or ethics. In some cases, this was combined with bedside teaching. Moreover, one of these universities was planning to offer an elective course for the first time in the summer semester of 2022 to discuss the new Dispositions of the Dying Act. Interviewees at one university reported that the courses were well attended, while those at the other university reported low participation. Too late awareness of palliative care in the main curriculum was cited as a possible reason.

#### Interprofessional teaching

In the questionnaire, six participants stated that palliative care education at their university partly took place in interprofessional collaboration with other professional groups. According to the interviews, interprofessional collaboration was mentioned in lectures at several universities. However, some interviewees claimed that this was not a suitable format for conveying interprofessional work in an illustrative way.

“[For medical school] I would like to think about: How do you get this interprofessional approach to be tangible and that it's enjoyable as well, and that eventually a complete and successful image can be conveyed.” (T 16, I 45).

“We can only ever say it, more or less strikingly: We have to work in a multi-professional, interdisciplinary way and we need the team and there are meetings here. But that is not practised for the students. […] However, it would be a very important part, especially because it would change the students' view a bit from this (raises fists) 'I am a doctor and I can do everything'.” (T 13, I 71–77).

At the time of the study courses that were organised, held or demonstrated by people from several professions took place at five universities. At one university, students had the chance to attend an interprofessional team meeting in a palliative care unit for a few minutes as part of mandatory practical training. Another university offered a seminar in cooperation with general medicine to introduce the Mobile Palliative Team (*Mobiles Palliativteam*). Here, team members from four different professions simulated a classic Mobile Palliative Team morning meeting in which a patient case was discussed. Students were then given the opportunity to guess the individual professions, discuss the case, and ask questions, which was always done with great interest on the part of the students. Other interprofessionally organised and taught courses mentioned were communication, introductory, and elective courses.

Whether interprofessional education should already take place at the undergraduate level was controversial. On the one hand, it was important to teach the basics mono-professionally so that students would later feel confident in medical practice. On the other hand, palliative care work required interprofessional teamwork, and students should be prepared for this at an early stage to become familiar with possible sources of tension and to reduce prejudices.

### Clinical practical year

The clinical practical year is a mandatory part of medical studies in Austria. It takes place in the sixth year of study, consists of 48 weeks and is divided into three tertials of equal length, of which one tertial must be dedicated to surgical and perioperative subjects, another tertial to internal medicine and the third tertial can be designed depending on the university [63].

**Fig. 2:**
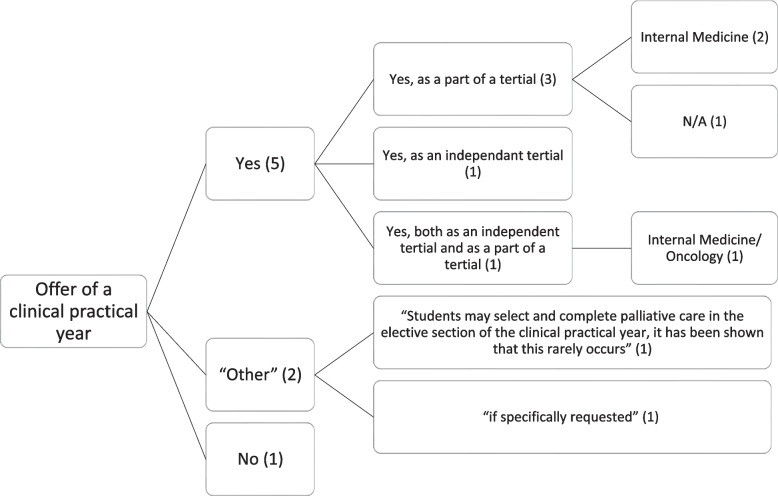
Offer of a clinical practical year at the universities

Figure 2 shows that, according to the questionnaire, at the time of the survey, it was possible at seven universities to complete at least part of a tertial of the clinical practical year in palliative care. At two of them it was stated that it was possible, but rarely happened or only occured "if explicitly requested."

In the interviews, the representatives of three universities said that a clinical practical year program was currently available. They added that it was popular and positively evaluated by the students but that there were only a few places available. At three universities, the interviewees reported that they wanted to work towards offering a clinical practical year soon.

At universities where it was not currently possible to offer a clinical practical year, the reasons given in the interviews were the lack of access to a palliative care unit (see Structures) and the university's unwillingness to recognise palliative care as part of the clinical internship year.

“There is no palliative care unit at the teaching hospital. […] [Instead], you can do an internship [in a hospice facility] voluntarily, you will of course get it recognised somehow, but they are not in the curriculum of the university. […] And this is not applicable for clinical practical year.” (T 13, I 83).

“I: In principle, does the university allow students to do the clinical practical year in palliative care units? – T 3: No, exactly. So that is prohibited so far” (T 3, I 184–185).

## Discussion

This is the first study to investigate palliative care in the undergraduate medical curriculum in Austria. Currently, palliative care is taught at seven universities, at another one the launch of a palliative care curriculum is planned in 2023. Overall, large differences were found in the number of hours, organisation, and teaching formats. Access to palliative care education in medical schools has not been regulated so far and does not meet the minimum standards recommended by the EAPC guidelines [32, 33]. Country-wide collaboration to improve the situation was felt to be insufficient by the participants. A reason for this could be that palliative care is perceived by many as terminal care (*Sterbemedizin*), offered in hospices but not at university clinics.

The teaching of palliative care in universities is heterogeneous. At universities offering mandatory palliative care teaching, the number of hours varies between two and 15 teaching units of 45 min. Thus, all universities are far from the recommended 40 teaching units of the EAPC curriculum of 2013, or the 72 h of training proposed by the EDUPALL project [32, 33]. This is not unusual in comparison to other European countries, as a diagram in the EAPC Atlas 2019 shows: Out of the 51 countries surveyed, there were only five countries with more than half of the universities teaching over 20 h of palliative care and 35 countries where none of the universities taught more than 20 h of palliative care [64]. However, the literature [32, 33, 65, 66], as well as the interviews in our study, caution not to overload the medical curriculum. Instead, palliative care content should also be addressed in other subjects in the sense of horizontal integration. Thus, students are frequently confronted with palliative care content without compromising the feasibility of medical studies.

The EAPC Recommendations suggest the following categories as teaching content for optimal palliative care education: basics of palliative care, pain and symptom management, psychosocial and spiritual aspects, ethical and legal issues, communication, and teamwork and self-reflection [32, 33]. The reported weighting of the proposed content varied from university to university, and in some cases, deviated considerably from the recommended distribution. Nevertheless, it can be positively highlighted that each of the six categories occurs in teaching at all universities, which was confirmed in the questionnaire and interviews.

According to a scoping review of 2022, the main focus of research on this topic in Austria to date has been on the number of universities where palliative care is taught as a mandatory subject [35]. The most recent data on this can be found in the 2019 EAPC Atlas, where data from 51 European countries are presented. There it was stated that in Austria, palliative care was taught as a mandatory subject at seven out of seven medical faculties [67]. According to our study, eight medical faculties exist, of which palliative care is a mandatory subject at six universities at the time of the survey. The difference in the data is most likely explained by the time and method of the survey. At one university, a bachelor's degree in human medicine was started for the first time in the winter semester of 2019/20, which could not yet be reflected in the 2019 EAPC Atlas. Furthermore, the data collected for the present study was provided by experts from all universities, whereas for the EAPC Atlas 2019, one central person gathered information for all Austrian universities. This leads to the assumption that this study provides more accurate data for Austria. A similar observation was made in a study on undergraduate palliative care education in Israel [44].

Literature search and interviews identified three palliative care chairs in Austria. Older sources mention one or two chairs [19, 68], which shows that the number has increased slightly. Nevertheless, many interviewees criticised that there had been too few efforts to establish chairs in Austria. Ideally, there should be palliative care chairs at universities whose holders would simultaneously run palliative care units and conduct palliative care research. This is consistent with a study by Gibbins, which postulates that promoting good palliative care teaching includes an ‘enthusiastic champion’ and the ‘availability of specialist palliative care units’. According to Gibbins, a chair is helpful but not essential for good university teaching if the palliative care ‘champion’ is well connected [69]. This statement has to be critically revised and rejected. Many participants in this study have been ‘champions’ for palliative care for years, struggling between the clinical duties, patient care and teaching at the university. It would be problematic to suggest that oncology, cardiology, neurology, or some other discipline is taught by a ‘champion ‘ who is only well connected, without having a chair or access to a clinic. Like other medical disciplines, palliative care requires structures to build a meaningful and sustainable curriculum for palliative care.

Teaching palliative care is a very special task, which requires ‘accepting the societal diversity and uniqueness of each patient and caregiver, [and] may frequently challenge the personal beliefs and values of both teachers and student’ [70]. Good clinical teachers are expected to ‘organise learning’, ‘knowing the field and state of the art’, ‘being ready to learn’, ‘teaching and leading by example’, and ‘being real’ [71]. Teachers need time, support, and training, which means that universities need to invest in their faculties to improve the situation [71, 72]. The lack of people to take over and continue ‘champion’s efforts has already been identified as a serious ‘bottle neck’ in countries that have been successful introducing palliative care into their undergraduate medical curricula [73].

Palliative care in Austria is taught primarily through lectures, seminars, small-group courses, practical training, and bedside teaching. Numerous studies recommend the combination of different teaching formats, especially active ones, to convey the content in a more sustainable and practice-oriented way and thus to take into account the different learning types among students [27, 32, 33, 44, 74–76]. Bedside teaching is suggested by the EAPC Recommendations and other literature as a valuable teaching format, but some challenges and rules need to be considered, which was also mentioned in the interviews [27, 32, 33, 69, 77–79]. In the case of concerns about patient well-being, an alternative to real patient contact was presented in a study by Hawkins, in which high-fidelity simulation also produced good results [80].

Our study found that examination of palliative care was predominantly a minor consideration. Both Gibbins and the EAPC Recommendations highlighted the value of assessment tools to consolidate what has been learned [32, 69].

Even though some Austrian universities presented interesting concepts in interprofessional teaching, the general impression was that, for the most part, hardly any courses were held in collaboration with several professional groups. While a Cochrane Review could not find evidence on interprofessional teaching [81], it is clearly recommended for palliative care in the EAPC Recommendations and other literature [24, 32, 71, 75, 82, 83]. Nevertheless, many interviewees emphasised that it should be taught in an illustrative and interactive way. This is already implemented in a very creative approach in some universities' mandatory teaching, while in others, it is only mentioned in lectures held by a person from the medical profession.

Networking and consultation were the recurring motifs in this study. The benefits of interdisciplinary teaching were emphasised not only by the interviewees in our study but also in many other studies, as well as the EAPC Recommendations [32, 33, 44, 75, 82, 84]. Moreover, it is necessary to ensure the horizontal integration of palliative care teaching. In this context, good communication between the different disciplines is essential to promote the expansion of high-quality interdisciplinary palliative care education [32, 44, 69].

Overall, the participants had different views on the best possible design of palliative care education and, with a few exceptions, were mostly poorly informed about the teaching offered at other universities. This shows that there is little consultation between key persons at different universities. Better communication for mutual support and curriculum optimisation would be beneficial.

However, many participants expressed their motivation to promote the expansion of undergraduate palliative care education. In Vienna, a scientific study on the use of medical comics in palliative care teaching was published, which showed predominantly positive experiences [85]. Publications such as this could help to establish new teaching formats and shift the focus on palliative teaching. Moreover, shortly after data collection, new research has revealed further positive developments. At the Paracelsus Medical University in Salzburg, a separate research institute for palliative care was founded [86] and according to a new state government program, an endowed professorship for palliative care is planned for the province of Tirol [87].

### Strengths and limitations

The data from the questionnaire and the interviews differed with regard to the number of universities with a chair, mandatory teaching, elective courses, and a clinical practical year. This could be due to data collection errors in both the questionnaire and interviews.

The questionnaire was tested several times; only those were recruited as key persons who were most knowledgeable according to the rector's and dean's offices of the universities, and all participants were made aware that the data would be anonymised. Nevertheless, it cannot be guaranteed that some answers may be inaccurate or incorrect due to misleading questions, lack of knowledge on the part of the participants, or social desirability.

In addition, it is possible that some aspects were not sufficiently addressed in the interviews and that certain information was not collected. This could be due to time constraints and the semi-structured design of the interviews, as well as the lack of experience of the interviewer. Furthermore, the thematic analysis of the interview material was probably subject to a certain degree of subjectivity since it was conducted with the support of the co-authors, but still mainly by the corresponding author.

Although the data collected in the questionnaire and interview could be partly inaccurate individually, the strength of the present study is that they are analysed and presented here in complement to each other. Thus, it can be assumed that the study as a whole is a meaningful reflection of reality.

Another strength of this study is its high participation rate, which gives our work a high representativity for the state of undergraduate palliative care education in Austria. The questionnaire was filled out by representatives from each university, and at least one person responsible for palliative care teaching per university was interviewed. Almost all university representatives were able to describe the overall concept of palliative care teaching at their university. The external persons were hardly informed about the current undergraduate education but outlined ideas for an optimal curriculum or better organisation of university teaching.

Due to the Corona pandemic, the study was planned so that participant contact and interviews were exclusively digital. Despite the distance caused by the video call instead of a face-to-face meeting, the conversational atmosphere of the interviews was mostly positively reported in the field notes.

## Conclusion

A mandatory palliative care program will be planned for all medical faculties in Austria for the next few years. At the time of the survey, it was established at most of the universities. Currently, few universities have a chair and some more universities have access to a palliative care unit. Overall, undergraduate palliative care education in Austria is heterogeneous. The number of hours of mandatory teaching varied. Lectures are provided at all universities, but some universities also integrate more active teaching formats, such as small-group courses and bedside teaching. Interprofessional and interdisciplinary teaching are aspired to in many places, but are not always sufficiently implemented. Better coordination and networking within and between universities would be beneficial for the expansion and quality of teaching.

### Supplementary Information

**Additional file 1.** 

**Additional file 2.**

## Data Availability

A translated version of the questionnaire and the interview guide can be found in the Additional Files. Further quotes from the interview transcripts and quotes on the original language are not published here to protect the confidentiality and anonymity of the participants. They are available on reasonable request from the corresponding author.

## Abbreviations

EAPC: European Association for Palliative Care
WHO: World Health Organization
T: Transcript
I: Item
n.d.: No date
h: Hours

## Footnotes

1 In some interviews there were connection problems with small interruptions and in one interview the participant could not activate their camera. Apart from that, all interviews could be conducted to the end without interruption.

2 Questions number 1 and number 3 as indicated in Additional File 2.

3 Questions number 19 and from number 20 to number 28 as indicated in Additional File 2.

4 Four of the key persons, who were only invited for an interview, did not give any feedback, or cancelled despite several contact attempts via mail or telephone. Reasons given for the cancellation were insufficient information about palliative care teaching at the university or lack of time for an interview.

## References

[1] Parlament der Republik Österreich: Hospiz- und Palliativfondsgesetz – HosPalFG. 2021. https://www.parlament.gv.at/PAKT/VHG/XXVII/ME/ME_00151/imfname_1006952.pdf. Accessed 20 July 2023.

[2] Parlament der Republik Österreich: Sterbeverfügungsgesetz – StVfG. 2021. https://www.parlament.gv.at/PAKT/VHG/XXVII/ME/ME_00150/imfname_1006947.pdf. Accessed 20 Jul 2023.

[3] Hospiz Österreich, Österreichische PalliativGesellschaft: Gut leben können und sterben dürfen. 2020.

[4] Hospiz Österreich, Österreichische PalliativGesellschaft: Gemeinsame Stellungnahme von Dachverband Hospiz Österreich und Österreichischer Palliativgesellschaft zum aktuellen Diskussionsprozess über die gesetzliche Regulierung des assistierten Suizids. 2021.

[5] World Health Organization: WHO Definition of Palliative Care 2002. 2002. https://www.dgpalliativmedizin.de/images/stories/pdf/WHO_Definition_2002_Palliative_Care_englisch-deutsch-2.pdf. Accessed 20 Jul 2023.

[6] Ahmedzai SH, Costa A, Blengini C, Bosch A, Sanz-Ortiz J, Ventafridda V, Verhagen SC (2004). Inter working Grp convened E: a new international framework for palliative care. Eur J Cancer.

[7] Fragemann K, Wiese C (2014). Pain education policies and initiatives in Europe. J Pain Palliat Care Pharmacother.

[8] Alt-Epping B: Interdisziplinarität und Multiprofessionalität. In: Palliativmedizin - das Skript. 7. Auflage edn Alt-Epping B, Nauck F (Eds.): Universitätsverlag Göttingen; 2019.

[9] Paal P, Lorenzl S, Schlömmer D (2021). Postgraduale Ausbildung im Bereich Palliative Care - Einblicke aus Salzburg. Universum Innere Medizin.

[10] Mückstein E, Aubauer G, Jarolim J, Huainigg F, Belakowitsch-Jenewein D, Scherak N, Vetter G: Bericht der parlamentarischen Enquete-Kommission zum Thema „Würde am Ende des Lebens“. 2015. https://www.parlament.gv.at/PAKT/VHG/XXV/A-HA/A-HA_00002_00344/index.shtml. Accessed 20 Jul 2023.

[11] World Health Assembly: Strengthening of palliative care as a component of comprehensive care throughout the life course. Resolution of May 24, 2014 at the 67th session of the World Health Assembly (WHA67.19). 2014.

[12] Arias N, Garralda E, De Lima L, Rhee JY, Centeno C (2019). Global palliative care and cross-national comparison: how is palliative care development assessed?. J Palliat Med.

[13] Centeno C, Garralda E, Carrasco JM (2017). den Herder-van der Eerden M, Aldridge M, Stevenson D, Meier DE, Hasselaar J: The Palliative Care Challenge: analysis of barriers and opportunities to integrate palliative care in Europe in the view of national associations. J Palliat Med.

[14] Lynch T, Clark D, Centeno C, Rocafort J, de Lima L, Filbet M, Hegedus K, Belle O, Giordano A, Guillén F, Wright M (2010). Barriers to the development of palliative care in Western Europe. Palliat Med.

[15] World Health Organization: Assessing the development of palliative care worldwide: a set of actionable indicators. 2021.

[16] Retschitzegger H: Spezialisierung in Palliativmedizin beschlossen. Österreichische PalliativGesellschaft. 2017. https://www.palliativ.at/aktuelles/nachrichten/news-detailseite/spezialisierung-in-palliativmedizin-beschlossen/. Accessed 20 Jul 2023.

[17] Österreichische Ärztekammer: Kundmachung der Österreichischen Ärztekammer Nr. 2/2017. 2017. https://www.aerztekammer.at/documents/261766/78341/Kundm+2017-2+1_Nov+SpezV+2017.pdf/af5afe3f-5ce7-6997-2bf1-3b9c54d822db. Accessed 20 Jul 2023.

[18] Hospiz Österreich: Übersicht der Lehrgänge Curriculum Level I, II und III. n.d. https://www.ulg-palliativecare.at/studium/info-curriculum-und-uebersicht. Accessed 20 July 2023.

[19] Hospiz Österreich: Bildung. n.d. https://www.hospiz.at/fachwelt/bildung/. Accessed 20 Jul 2023.

[20] Köstenberger M, Neuwersch S, Weixler D, Pipam W, Zink M, Likar R (2019). Prevalence of palliative care patients in emergency departments. Wien Klin Wochenschr.

[21] Hackl M, Ihle P (2022). Krebserkrankungen in Österreich.

[22] STATISTIK AUSTRIA Bundesanstalt Statistik Österreich: Demographisches Jahrbuch. Wien 2022.

[23] Bowden J, Dempsey K, Boyd K, Fallon M, Murray SA (2013). Are newly qualified doctors prepared to provide supportive and end-of-life care? a survey of Foundation Year 1 doctors and consultants. J R Coll Physicians Edinb.

[24] Dobrowolska B, Mazur E, Pilewska-Kozak A, Dońka K, Kosicka B, Palese A (2019). Predicted difficulties, educational needs, and interest in working in end of life care among nursing and medical students. Nurse Educ Today.

[25] Gibbins J, McCoubrie R, Forbes K (2011). Why are newly qualified doctors unprepared to care for patients at the end of life?. Med Educ.

[26] Centeno C, Ballesteros M, Carrasco JM, Arantzamendi M (2016). Does palliative care education matter to medical students? The experience of attending an undergraduate course in palliative care. BMJ Support Palliat Care.

[27] Centeno C, Rodríguez-Núñez A (2015). The contribution of undergraduate palliative care education: does it influence the clinical patient's care?. Curr Opin Support Palliat Care.

[28] Fortin Magaña M, Diaz S, Salazar-Colocho P, Feng A, López-Saca M: Long-term effects of an undergraduate palliative care course: a prospective cohort study in El Salvador. BMJ Support Palliat Care 2020. 10.1136/bmjspcare-2020-002311 33219104

[29] Fraser HC, Kutner JS, Pfeifer MP (2001). Senior medical students' perceptions of the adequacy of education on end-of-life issues. J Palliat Med.

[30] Gerlach C, Mai S, Schmidtmann I, Massen C, Reinholz U, Laufenberg-Feldmann R, Weber M (2015). Does interdisciplinary and multiprofessional undergraduate education increase students' self-confidence and knowledge toward palliative care? Evaluation of an undergraduate curriculum design for palliative care at a German academic hospital. J Palliat Med.

[31] Mutto EM, Cantoni MN, Rabhansl MM, Villar MJ (2012). A perspective of end-of-life care education in undergraduate medical and nursing students in Buenos Aires. Argentina J Palliat Med.

[32] Elsner F, Centeno C, Cetto G, De Conno F, Ellershaw J, Eychmuller S, Filbet M, Larkin P, Mason S: Recommendations of the European Association for Palliative Care (EAPC) for the Development of Undergraduate Curricula in Palliative Medicine at European Medical Schools. Report of the EAPC Steering Group on Medical Education and Training in Palliative Care. European Association for Palliative Care. 2013.

[33] Mason SR, Ling JL, Stanciulescu L, Payne C, Paal P, Albu S, Noguera A, Boeriu E, Poroch V, Elsner F, Mosoiu D (2020). From European association for palliative care recommendations to a blended, standardized, free-to-access undergraduate curriculum in palliative medicine: The EDUPALL Project. J Palliat Med.

[34] Filbet M, Centeno C, De Conno F, Ellershaw J, Elsner F, Eychmuller S, Kaasa S, Larkin P: Curriculum in Palliative Care for Undergraduate Medical Education: Recommendations of the European Association for Palliative Care. EAPC. 2007.

[35] Toussaint V, Elsner F, Paal P: Entwicklung der universitären palliativmedizinischen Lehre in Österreich: ein Scoping Review. Z Palliativmed 2022.

[36] Kettles AM, Creswell JW, Zhang W (2011). Mixed methods research in mental health nursing. J Psychiatr Ment Health Nurs.

[37] Bogner A, Littig B, Menz W: Introduction: Expert Interviews — An Introduction to a New Methodological Debate. In: Interviewing experts. Basingstoke England: Palgrave Macmillan; 2009: 1–13.

[38] Malterud K, Siersma VD, Guassora AD (2016). Sample size in qualitative interview studies: guided by information power. Qual Health Res.

[39] Cristancho SM, Goldszmidt M, Lingard L, Watling C (2018). Qualitative research essentials for medical education. Singapore Med J.

[40] Willemsen AM, Paal P, Zhang S, Mason S, Elsner F (2021). Chinese medical teachers' cultural attitudes influence palliative care education: a qualitative study. BMC Palliat Care.

[41] Kallio H, Pietilä AM, Johnson M, Kangasniemi M (2016). Systematic methodological review: developing a framework for a qualitative semi-structured interview guide. J Adv Nurs.

[42] Eysenbach G (2004). Improving the quality of Web surveys: the Checklist for Reporting Results of Internet E-Surveys (CHERRIES). J Med Internet Res.

[43] Ohlmeier L, Scherg A, Ilse B, Elsner F (2021). Stand der palliativmedizinischen Lehre in Deutschland: Bestandsaufnahme an den medizinischen Fakultäten im Jahr 2018. Der Schmerz.

[44] Elsner F, Müller A, Gil W, Paal P: "The education is a mirror of where palliative care stands in Israel today": An exploration of palliative care undergraduate education at medical schools in Israel. Palliat Support Care 2021:1–8. 10.1017/S1478951521001450 34503603

[45] SoSci Survey GmbH: SoSci Survey – the Solution for Professional Online Questionnaires. n.d. https://www.soscisurvey.de/en/index. Accessed 20 Jul 2023.

[46] VERBI Software GmbH: MAXQDA Analytics Pro. n.d. https://www.maxqda.com/products/maxqda-analytics-pro. Accessed 20 Jul 2023.

[47] Braun V, Clarke V (2006). Using thematic analysis in psychology. Qual Res Psychol.

[48] Schoonenboom J: Developing the Meta-Inference in Mixed Methods Research through Successive Integration of Claims. In: The Routledge Handbook for Advancing Integration in Mixed Methods Research. Hitchcock JH, Onwuegbuzie AJ (Eds.): Taylor & Francis; 2022: 55–70.

[49] Feustel R: Transkription. n.d. https://home.uni-leipzig.de/methodenportal/transkription/. Accessed 20 Jul 2023.

[50] O'Brien BC, Harris IB, Beckman TJ, Reed DA, Cook DA (2014). Standards for reporting qualitative research: a synthesis of recommendations. Acad Med.

[51] Tong A, Sainsbury P, Craig J (2007). Consolidated criteria for reporting qualitative research (COREQ): a 32-item checklist for interviews and focus groups. Int J Qual Health Care.

[52] Medizinische Universität Wien: Weltspitze seit Jahrhunderten: Die Geschichte der MedUni Wien. n.d. https://www.meduniwien.ac.at/web/ueber-uns/geschichte/. Accessed 20 Jul 2023.

[53] Medizinische Universität Innsbruck: Zahlen, Daten, Fakten. Medizinische Universität Innsbruck. 2020. https://www.i-med.ac.at/pr/docs/Zahlen_Daten_Fakten_2020.pdf. Accessed 20 Jul 2023.

[54] Medizinische Universität Graz: Ein Streifzug durch die Geschichte. n.d. https://www.medunigraz.at/geschichte. Accessed 20 Jul 2023.

[55] Paracelsus Medizinische Privatuniversität: Paracelsus Universität feierte 20-jähriges Gründungsjubiläum. 2022. https://www.pmu.ac.at/news/article/paracelsus-universitaet-feierte-20-jaehriges-jubilaeum.html. Accessed 20 Jul 2023.

[56] Karl Landsteiner Privatuniversität für Gesundheitswissenschaften GmbH: 10 Jahre KL. 2023. https://www.kl.ac.at/de/universitaet/ueber-uns/10-jahre-kl. Accessed 20 Jul 2023.

[57] Johannes Kepler Universität Linz: Die Johannes Kepler Universität. Eine lange und bewegte Geschichte. n.d. https://www.jku.at/die-jku/ueber-uns/geschichte/. Accessed 20 Jul 2023.

[58] Sigmund Freud PrivatUniversität Wien: Geschichte. n.d. https://www.sfu.ac.at/de/ueber-sfu/geschichte/. Accessed 20 Jul 2023.

[59] Müßig D: Jahresbericht 2019. Danube Private University. 2020. https://www.dp-uni.ac.at/admin/filemanager/userfiles/2019_JB.pdf. Accessed 20 Jul 2023.

[60] Medizinische Universität Wien: Eva Katharina Masel übernimmt Professur für Palliativmedizin. 2022. https://www.meduniwien.ac.at/web/ueber-uns/news/news-im-jaenner-2022/eva-katharina-masel-uebernimmt-professur-fuer-palliativmedizin/. Accessed 20 Jul 2023.

[61] Paracelsus Medizinische Privatuniversität: Abschluss des 9. Universitätslehrgangs Palliativ Care an der Paracelsus Universität in Salzburg. 2018. https://www.pmu.ac.at/news/article/abschluss-des-universitaetslehrgangs-palliativ-care-1.html. Accessed 20 Jul 2023.

[62] Gesellschaft der Ärzte in Wien - Billrothhaus: Antrittsvorlesungen der Sigmund Freud Privatuniversität Wien (SFU). 2019. https://billrothhaus.at/index.php?option=com_vf_veranstaltungskalender&task=lecture&limitstart=9&veID=1500. Accessed 20 Jul 2023.

[63] Rechnungshof Österreich: Ärzteausbildung Bericht des Rechnungshofes. 2021. https://www.parlament.gv.at/dokument/XXVII/III/501/imfname_1024802.pdf. Accessed 20 Jul 2023.

[64] Arias-Casais N, Garralda E, Rhee J, de Lima L, Pons J, Clark D, Hasselaar J, Ling J, Mosoiu D, Centeno C: Development and Integration of Palliative Care across Europe: Palliative medicine education across Europe. In: EAPC Atlas of Palliative Care in Europe 2019 Vilvoorde: EAPC Press; 2019: 61–64.

[65] Horowitz R, Gramling R, Quill T (2014). Palliative care education in U.S. medical schools. Med Educ.

[66] Linklater GT, Bowden J, Pope L, McFatter F, Hutchison SM, Carragher PJ, Walley J, Fallon M, Murray SA (2014). Developing learning outcomes for medical students and foundation doctors in palliative care: a national consensus-seeking initiative in Scotland. Med Teach.

[67] Arias-Casais N, Garralda E, Rhee J, de Lima L, Pons J, Clark D, Hasselaar J, Ling J, Mosoiu D, Centeno C: Development of Palliative Care at the Country-Level: Austria. In: EAPC Atlas of Palliative Care in Europe 2019 Vilvoorde: EAPC Press; 2019: 100, 101.

[68] Centeno C L, Donea O, Rocafort J, Clarck D.: EAPC Atlas of Palliative Care in Europe 2013 - Full Edition. Milan: EAPC Press: EAPC; 2013.

[69] Gibbins J, McCoubrie R, Maher J, Forbes K (2009). Incorporating palliative care into undergraduate curricula: lessons for curriculum development. Med Educ.

[70] Paal P: Hurdle Hopping: collaborating to overcome challenges in palliative care education. EAPC Blog. 2023. https://eapcnet.wordpress.com/2023/02/28/hurdle-hopping-collaborating-to-overcome-challenges-in-palliative-care-education/. Accessed 20 Jul 2023.

[71] Paal P, Brandstötter C, Lorenzl S, Larkin P, Elsner F (2019). Postgraduate palliative care education for all healthcare providers in Europe: Results from an EAPC survey. Palliat Support Care.

[72] Noguera A, Mosoiu D, Payne C, Paal P (2022). "This Project Helped Me to Grow": Experiences of Teaching Palliative Care Using the EDUPALL Project Medical Undergraduate Curriculum. J Palliat Med.

[73] Paal P, Brandstötter C, Elsner F, Lorenzl S, Osterbrink J, Stähli A: European interprofessional postgraduate curriculum in palliative care: A narrative synthesis of field interviews in the region of Middle, Eastern, and Southeastern Europe and Central and West Asia. Palliat Support Care 2022:1–10. 10.1017/S1478951522001651 36545761

[74] Hökkä M, Rajala M, Kaakinen P, Lehto JT, Pesonen HM (2022). The effect of teaching methods in palliative care education for undergraduate nursing and medical students: a systematic review. Int J Palliat Nurs.

[75] Afshar K, Matthias K, Paulmann V, Engel B, Stiel S, Schneider N (2020). Medical training in palliative care at the Hannover Medical School: development of the interdisciplinary and cross-sectoral education in the cross-sectional subject Q13 (WEISE-Q13). Schmerz.

[76] Hildebrandt J, Ilse B, Schiessl C (2013). "Traumcurriculum" - Wünsche Medizinstudierender an die Ausbildung in Palliativmedizin. Palliativmed.

[77] Noguera A, Bolognesi D, Garralda E, Beccaro M, Kotlinska-Lemieszek A, Furst CJ, Ellershaw J, Elsner F, Csikos A, Filbet M, Biasco G, Centeno C (2018). How do experienced professors teach palliative medicine in European Universities? A cross-case analysis of eight undergraduate educational programs. J Palliat Med.

[78] Harris DG (2011). Overcoming the challenges of bedside teaching in the palliative care setting. BMJ Support Palliat Care.

[79] Kriesen U, Altiner A, Müller-Hilke B (2018). Perception of bedside teaching within the palliative care setting-views from patients, students and staff members. Ann Palliat Med.

[80] Hawkins A, Tredgett K (2016). Use of high-fidelity simulation to improve communication skills regarding death and dying: a qualitative study. BMJ Support Palliat Care.

[81] Reeves S, Perrier L, Goldman J, Freeth D, Zwarenstein M (2013). Interprofessional education: effects on professional practice and healthcare outcomes (update). Cochrane Database Syst Rev.

[82] Friesen L, Andersen E (2019). Outcomes of collaborative and interdisciplinary palliative education for health care assistants: a qualitative metasummary. J Nurs Manag.

[83] Pahor M, Rasmussen BH (2009). How does culture show? a case study of an international and interprofessional course in palliative care. J Interprof Care.

[84] Head BA, Schapmire T, Hermann C, Earnshaw L, Faul A, Jones C, Kayser K, Martin A, Shaw MA, Woggon F, Pfeifer M (2014). The Interdisciplinary Curriculum for Oncology Palliative Care Education (iCOPE): meeting the challenge of interprofessional education. J Palliat Med.

[85] Adamidis F, Kum L, Kitta A, Unseld M, Praschinger A, Koblizek R, Anvari-Pirsch A, Kutalek R, Melichar P, Zeilinger EL, Masel EK (2022). The potential of medical comics to teach palliative care skills: a cross-sectional study of 668 medical students. Ann Palliat Med.

[86] Paracelsus Medizinische Privatuniversität: Institut für Palliative Care n.d. https://www.pmu.ac.at/institute-kliniken/universitaetsinstitute/palliative-care.html. Accessed 20 Jul 2023.

[87] Tiroler Volkspartei und SPÖ Tirol: Stabilität in der Krise. Erneuerung für Tirol. Regierungsprogramm für Tirol 2022 - 2027. 2022. https://www.tirol.gv.at/fileadmin/bilder/navigation/regierung/2022/Regierungsprogramm_2022_Stabilitaet_Erneuerung.pdf. Accessed 20 Jul 2023.

