# Supplementary material for: The state of undergraduate palliative care education at Austrian medical schools – a mixed methods study
Source: BMC Palliat Care. 2023 Oct 10;22:151. doi: 10.1186/s12904-023-01255-9 (PMC10563205; doi:10.1186/s12904-023-01255-9)
Supplement: Supplementary file 1 — Additional file 1. [file 12904_2023_1255_MOESM1_ESM.pdf]

The State of undergraduate Palliative care Education at Austrian Medical Schools – a mixed Methods Study

Interview guide PallAustria22 (translated version)

Key questions

Between three and four key questions were emailed to participants a few days before the interview along with the invitation link for the Zoom meeting. This allowed participants to prepare for the interview. In addition, the same email included a remark that participants would have the opportunity in the interview to speak freely and openly about any aspects that they felt were important to them about the topic of undergraduate palliative care education.

Key questions for the university representatives with current occupation in undergraduate palliative care education

- What is your role in palliative care education at your university?
- How would you describe the palliative care education of medical students at your university?
- What factors influence palliative care education at your university in particular and in Austria in general?

Alternative key questions for the external participants and university representatives with former occupation in undergraduate palliative care education

- What has been the development of palliative care education at your university?
- To what extent are you or have you been involved in undergraduate palliative care education in Austria?
- What are your impressions of undergraduate palliative care education in Austria?
- What do you think influences undergraduate palliative care education in Austria?
- In your opinion, how will undergraduate palliative care education in Austria develop in the future?
- To what extent is postgraduate palliative care education relevant for young physicians?
- What are the contents of postgraduate palliative care education?
- To what extent were physicians you supervised prepared for palliative care?
- How would you envision good university palliative care teaching?

Bonus question

The bonus question was not sent in advance but was always asked at the end of the interview to give participants another opportunity to speak openly about all their ideas on palliative care education and to return to or elaborate on individual points already raised in the interview.

- What does palliative care education mean to you?

Semi-structured interview guide

This interview guide was not intended to impose a strict sequence, but rather served as a guide for the interviewer to cover the most important topics. The order and selection of the questions as well as the wording of the questions were flexibly adapted to the respective course of the interview.

1. Could you please describe your **role** in palliative care education at your university?

- What **position** do you hold at your university?
- What palliative care **courses** do you teach at your university?
- How much **time** do you spend on palliative care teaching?
- **Since when** do you hold your teaching position at your university?

2. How is **palliative care education** organised at your university?
- Could you please describe which **palliative care courses** are offered at your university?
- Can you assess how **intensive** palliative care teaching is at your university?
- **How much time**, approximately, does palliative care education occupy in your university's medical curriculum?
- In which **semester** are palliative care courses offered at your university?
- To what extent is palliative care education **interprofessional** at your university?
- What palliative care **contents** are primarily **taught** at your university?
- What palliative care **contents** are primarily **examined** at your university?

3. How would you **evaluate** palliative care education at your university?
- Is there anything you would **like to do differently** concerning palliative care education at your university?
- What do you think are the main priorities of **good palliative care education?**
- In your opinion, how can the little time left in the medical curriculum for palliative care education be used in the **best possible way** to prepare students for the care of patients with life-threatening illness?
- In your experience, what is the best **time** to offer palliative care education? (in which semester)
- Do the **students** at your university have the opportunity to **evaluate** palliative care education?
  - If yes:
    - What is the **outcome** of this evaluation?
    - Are students **interested** in palliative care education? How does this become noticeable?
    - Is there anything in particular that students **would like** to have **more** of?
  - If no: Can you estimate, …
    - …**how** palliative care education is received by the students at your university?
    - …whether the students at your university are **interested** in palliative care?
    - …whether the students at your university **would like** to have something **more** of?

4. What has been the **development** of palliative care education at your university?
- What were the **beginnings** of palliative care education at your university?
- What has **changed** since then?
- What has **guided** palliative care education at you university when it was first established?

5. What **influences** palliative care education at your university/in Austria in general?
- To what extent is palliative care education at your university/in Austria in general **supported** externally?
- What other forms of support would be **helpful as well** for improving palliative care education at your university/in Austria in general?

6. What do you think is **next** for the palliative care education at your university/in Austria in general?
- What are the **challenges** ahead for palliative care teaching?
- What are your **hopes** for palliative care teaching?

7. What does palliative care education **mean** to you?
- **Why** is palliative care education **important** in your eyes?
- What is your **relationship** towards palliative care education?
- To what extent has palliative care played a **role in your education**?
- What have you found **lacking** in your own palliative care education?
