# Supplementary material for: The state of undergraduate palliative care education at Austrian medical schools – a mixed methods study
Source: BMC Palliat Care. 2023 Oct 10;22:151. doi: 10.1186/s12904-023-01255-9 (PMC10563205; doi:10.1186/s12904-023-01255-9)
Supplement: Supplementary file 2 — Additional file 2. [file 12904_2023_1255_MOESM2_ESM.pdf]

The State of undergraduate Palliative care Education at Austrian Medical Schools – a mixed Methods Study

Questionnaire (translated version)

Notes to this document

This document is designed to simplify the answering of the questions and to provide guidance for a better orientation and possible consultation with colleagues. Please enter your answers directly in digital form. The link for this digital questionnaire was sent to you by e-mail. If you did not receive a link, please contact one of the contact persons.

Please note that in the digital format of the questionnaire so-called filters were used and therefore the numbering of the digital questionnaire may differ from that of this analog questionnaire. The filters show or hide later questions or topic blocks depending on the answer to a question. For example, the questions 10 to 19 are only visible if you have answered question 8 with "Yes". If you answered "No" to question 8, these questions are skipped and you continue directly with question 20, which then has the number 10.

Below is the questionnaire on the "The State of undergraduate Palliative care Education at Austrian Medical Schools". The questions and answer options correspond exactly to those you will find in the digital questionnaire. The consent form is the first page of the digital questionnaire. To participate in the questionnaire, you must agree to the consent form.

Informed consent

Dear Sir or Madam,

we thank you very much for your participation in the study ***The State of undergraduate Palliative care Education at Austrian Medical Schools***. The aim of this questionnaire is to record and document the current teaching of palliative care at the individual medical faculties of all Austrian universities. It is intended that only ***one key person*** in palliative care education from each university will complete the questionnaire to avoid any duplication. This key person can, of course, consult with colleagues at the same university if there is any uncertainty.

The questionnaire takes **15-20 minutes to complete**. Within the questionnaire, it is always possible to return to questions that have already been completed. Individual questions can be skipped. Only the questions about whether palliative care is offered as a mandatory or elective subject at your university must be answered for further completion of the questionnaire. The processing of the questionnaire can be interrupted at any time and resumed at a later time by following the instructions of *SoSci Survey*.

Please answer the questions as truthfully as possible on behalf of the university you represent. Please refer to the **winter semester 2021/22 and the summer semester 2022**.

It is planned to repeat the survey of palliative care education at regular intervals over the next few years, capturing developments in the teaching of palliative care.

The data collected will be anonymized; it will not be possible to draw conclusions about the individual participants or universities. The data you provide will be published in a medical journal and may serve as a basis for further studies in the future. Your actual identity and personal data will be kept ***confidential*** in accordance with the applicable data protection law and will not be shared with third parties at any time during the study. We will not use your name or any information that could identify you in any publications or presentations.

Participation in the study is ***voluntary*** and can be discontinued at any time. Refusal or discontinuation of participation in the study does not imply any disadvantages for the invited persons.

If you have any questions or encounter any problems while completing the questionnaire, please feel free to contact the people listed below.

Thank you for taking the time to support our research!

- Cand. med. **Véronique Toussaint** (Department of Palliative Medicine, Medical Faculty RWTH Aachen University): veronique.toussaint@rwth-aachen.de
- Prof. Dr. **Frank Elsner** (Department of Palliative Medicine, Medical Faculty RWTH Aachen University): felsner@ukaachen.de
- Prof. Dr. **Piret Paal** (Institute of Palliative Care, Paracelsus Medical University): piret.paal@pmu.ac.at
- **Rainer Simader** (Hospiz Österreich, Österreichische Palliativgesellschaft): rainer.simader@hospiz.at

I hereby acknowledge that I have read and understand the informed consent and agree to participate in this questionnaire.

- Yes
- No (do not participate in this study)

Information about yourself

1. **What is your role in your university?**
    - Assistent Professor
    - Associate Professor
    - University Professor
    - Other: *(please specify)*

2. **Which professional group do you belong to?**
   Multiple answers possible.
    - Physicians
    - Nursing
    - Psychology
    - Other: *(please specify)*

3. **If you are a physician, to which specialty do you belong?**
   Multiple answers possible.
    - Anesthesia
    - Neurology
    - Oncology
    - Internal medicine
    - General medicine
    - Other: *(please specify)*

4. **What qualifications have you acquired in palliative care?**
   Multiple answers possible.
    - None
    - Further qualification in Palliative care (ÖÄK-diploma)
    - Additional diploma in palliative care (Palliativbasislehrgang)
    - Master of Science in Palliative care (Universitätslehrgang Palliative care)
    - Other: *(please specify)*

General information on the palliative care education at your university

5. **Is there a professorship or chair of palliative care at your university?**
   Single choice. The option "Additional remarks" can be ticked additionally.
    - Yes, there is a chair for palliative care.
    - Yes, there is a professorship for palliative care.
    - No
    - Additional remarks: *(please specify)*

6. **Which structures or institutions are involved in the teaching of palliative care at your university?**
   Multiple answers possible.
    - Palliative care ward in the affiliated university hospital
    - Palliative care ward in a teaching hospital of the university
    - Hospice
    - Mobile palliative team
    - Other: *(please specify)*
    - None

7. **Does palliative care education at your university partly take place in interprofessional collaboration?**
   Multiple answers possible.
   - Yes, as part of the mandatory curriculum with the involvement of the following professional groups: *(please specify)*
   - Yes, as part of the elective curriculum in palliative care with the involvement of the following professional groups: *(please specify)*
   - No
   - Additional remarks: *(please specify)*

8. **Is palliative care a mandatory subject at your university?**
   Single choice. The option "Additional remarks" can be ticked additionally.
   - Yes
   - No
   - Additional remarks: *(please specify)*

   Note: If you ticked "Yes" to this question, detailed questions about teaching palliative care as a **mandatory subject** at your university will be unlocked below. If you answered "No" to this question, this section will be skipped.

9. **Are courses in palliative care offered as elective courses outside the mandatory curriculum at your university?**
   Single choice. The option "Additional remarks" can be ticked additionally.
   - Yes
   - No
   - Additional remarks: *(please specify)*

   Note: If you ticked "Yes" to this question, detailed questions about teaching palliative care as an **elective subject** at your university will be unlocked below. If you answered "No" to this question, this section will be skipped.

About the teaching of palliative care as a <u>mandatory</u> subject at your university

10. **How is palliative care taught as a mandatory subject at your university?**
    Single choice. The option "Additional remarks" can be ticked additionally.
    - As an independent subject
    - As part of another discipline: *(please specify)*
    - Additional remarks: *(please specify)*

11. **Since when has palliative care been taught as a mandatory subject at your university?**
    Single choice. The option "Additional remarks" can be ticked additionally.
    - Since less than 5 years
    - Since 5 to 10 years
    - Since more than 10 years
    - Additional remarks: *(please specify)*

12. **How many teaching units (45 minutes each) does the education of palliative care consist of as a mandatory subject at your university?**
    Single choice. The option "Additional remarks" can be ticked additionally.
    - 1-10
    - 11-20
    - 21-30
    - 31-40
    - 41-50
    - 51-60
    - >60
    - Additional remarks: *(please specify)*

13. **In which years of study is palliative care scheduled as a mandatory subject in the curriculum of your university?**
    Multiple answers possible.
    - 1st year
    - 2nd year
    - 3rd year
    - 4th year
    - 5th year
    - Additional remarks: *(please specify)*

14. **As a mandatory subject, into what contents is the education of palliative care at your university divided and how high would you estimate the weighting of the respective parts in percent?**
    Please enter a percentage (between 0 and 100) for each content and distribute these percentages so that the sum is 100%.
    - Basics of palliative care *(enter percentage)* %
    - Pain and symptom control *(enter percentage)* %
    - Psychosocial and spiritual aspects *(enter percentage)* %
    - Ethical and legal issues *(enter percentage)* %
    - Communication *(enter percentage)* %
    - Teamwork and self-reflection *(enter percentage)* %
    - Other: *(please specify)* *(enter percentage)* %

15. **What teaching formats are used in the education of palliative care as a mandatory subject at your university?**
    Multiple answers possible.
    - Lecture
    - Seminar
    - Practical training
    - Bedside teaching
    - E-Learning
    - Training with simulation patients
    - Problem-based learning
    - Case-based learning
    - Patient presentation
    - Other: *(please specify)*

16. **Which examination formats are used in the education of palliative care as a mandatory subject at your university?**
Multiple answers possible.
    - Written exam with multiple choice
    - Written exam with free text
    - OSCE („objective structured clinical examination“)
    - Oral exam
    - Clinical evaluation exercise (Mini-CEX)
    - Self-assessment questionnaire (SAQ)
    - Thesis
    - Reflection essay
    - Other: *(please specify)*
    - None

17. **Which areas are primarily addressed in the education of palliative care as a mandatory subject at your university?**
Multiple answers possible.
    - Knowledge (Cognitive learning objectives)
    - Skills (Psychomotoric learning objectives)
    - Attitude (Affective learning objectives)
    - Additional remarks: *(please specify)*

18. **Which areas are primarily examined in the education of palliative care as a mandatory subject at your university?**
Multiple answers possible.
    - Knowledge (Cognitive learning objectives)
    - Skills (Psychomotoric learning objectives)
    - Attitude (Affective learning objectives)
    - Additional remarks: *(please specify)*

19. **Several organizations and institutions have published recommendations for a mandatory palliative care curriculum. Which recommendations are used as guidelines for the education of palliative care as a mandatory subject at your university?**
Please specify the organizations or institutions that have published recommendations relevant to your university. You are welcome to add further details to your information, such as the title of the recommendations or the year of publication.
    - *(please specify)*

About the teaching of palliative care as an elective subject at your university

20. How is palliative care taught as an elective subject at your university?
Single choice. The option "Additional remarks" can be ticked additionally.

- As an independent subject
- As part of another discipline: *(please specify)*
- Additional remarks: *(please specify)*

21. Since when has palliative care been taught as an elective subject at your university?
Single choice. The option "Additional remarks" can be ticked additionally.

- Since less than 5 years
- Since 5 to 10 years
- Since more than 10 years
- Additional remarks: *(please specify)*

22. How many teaching units (45 minutes each) does the education of palliative care consist of as an elective subject at your university?
Single choice. The option "Additional remarks" can be ticked additionally.

- 1-10
- 11-20
- 21-30
- 31-40
- 41-50
- 51-60
- >60
- Additional remarks: *(please specify)*

23. In which years of study is palliative care scheduled as an elective subject in the curriculum of your university?
Multiple answers possible.

- 1st year
- 2nd year
- 3rd year
- 4th year
- 5th year
- Additional remarks: *(please specify)*

24. As an elective subject, into what contents is the education of palliative care at your university divided and how high would you estimate the weighting of the respective parts in percent?
Please enter a percentage (between 0 and 100) for each content and distribute these percentages so that the sum is 100%.

- Basics of palliative care *(enter percentage)* %
- Pain and symptom control *(enter percentage)* %
- Psychosocial and spiritual aspects *(enter percentage)* %
- Ethical and legal issues *(enter percentage)* %
- Communication *(enter percentage)* %
- Teamwork and self-reflection *(enter percentage)* %
- Other: *(please specify)* *(enter percentage)* %

25. **What teaching formats are used in the education of palliative care as an elective subject at your university?**
    Multiple answers possible.
    - Lecture
    - Seminar
    - Practical training
    - Bedside teaching
    - E-Learning
    - Training with simulation patients
    - Problem-based learning
    - Case-based learning
    - Patient presentation
    - Other: *(please specify)*

26. **Which examination formats are used in the education of palliative care as an elective subject at your university?**
    Multiple answers possible.
    - Written exam with multiple choice
    - Written exam with free text
    - OSCE („objective structured clinical examination“)
    - Oral exam
    - Clinical evaluation exercise (Mini-CEX)
    - Self-assessment questionnaire (SAQ)
    - Thesis
    - Reflection essay
    - Other: *(please specify)*
    - None

27. **Which areas are primarily addressed in the education of palliative care as an elective subject at your university?**
    Multiple answers possible.
    - Knowledge (Cognitive learning objectives)
    - Skills (Psychomotoric learning objectives)
    - Attitude (Affective learning objectives)
    - Additional remarks: *(please specify)*

28. **Which areas are primarily examined in the education of palliative care as an elective subject at your university?**
    Multiple answers possible.
    - Knowledge (Cognitive learning objectives)
    - Skills (Psychomotoric learning objectives)
    - Attitude (Affective learning objectives)
    - Additional remarks: *(please specify)*

About the clinical practical year

29. Do medical students have the opportunity to complete a section of the Clinical Practical Year in palliative care at the university hospital or at a teaching hospital of your university?
Multiple answers possible.

- Yes, as a part of a tertial: *(please specify)*
- Yes, as an independent tertial
- No
- Additional remarks: *(please specify)*
